# Prediction model for intrapartum cesarean delivery among women with gestational diabetes mellitus

**DOI:** 10.1007/s00404-025-08147-8

**Published:** 2025-08-12

**Authors:** Itamar Gilboa, Daniel Gabbai, Emmanuel Attali, Liran Hiersch, Anat Lavie, Yariv Yogev

**Affiliations:** 1 Department of Obstetrics and Gynecology, Lis Hospital for Women’s Health, Tel Aviv Sourasky Medical Center, 6 Weizmann St, 6423906 Tel Aviv, Israel; 2 Gray Faculty of Medicine, Tel Aviv University, Tel Aviv, Israel

**Keywords:** Gestational diabetes, Intrapartum cesarean delivery, Labor, Prediction model

## Abstract

**Purpose:**

To identify risk factors and to develop a predictive model for cesarean delivery (CD) in women with gestational diabetes mellitus (GDM).

**Study design:**

A retrospective cohort study, in a single university-affiliated tertiary medical center, was performed. All women with GDM and a singleton pregnancy who had a trial of labor between 2011 and 2023 were included. Women who chose an elective CD, those with pre-gestational diabetes, estimated fetal weight ≥ 4000 g, previous CD, and those with non-viable fetuses were excluded. Maternal characteristics of women who delivered vaginally were compared to those who underwent an intrapartum CD. Factors associated with CD were examined using univariate and multivariate analysis. A score was developed to predict the need for intrapartum CD. A receiver operating characteristic curve (ROC) was utilized for the model. Internal validation was performed using a 70/30 train-test split, with model performance evaluated on the validation set using ROC analysis. The main outcome was an unplanned intrapartum CD.

**Results:**

Overall, 11,305 women were included; of them 676 (6.0%) underwent intrapartum CD. Several risk factors were identified, including maternal age ≥ 40 years, body mass index > 30kg/m^2^, maternal height < 1.6m, in vitro fertilization, gestational weight gain > 15kg, nulliparity, induction of labor, oxytocin use during labor, preeclampsia, meconium-stained amniotic fluid, and birthweight ≥ 3,500g. Pharmacologically treated GDM was not associated as a risk factor. Epidural anesthesia was associated with reduced risk for intrapartum CD. A prediction score model has reached a predictive performance with an AUC of 0.807 (95%CI 0.79–0.83, *p* < 0.001). On internal validation using the 30% hold-out cohort, the model maintained strong performance with an AUC of 0.788 (95% CI 0.76–0.82).

**Conclusion:**

Several maternal and intrapartum factors were associated with intrapartum cesarean delivery in women with GDM. A prediction model based on these factors may help identify high-risk patients and support delivery planning.

## Introduction

The timing and mode of delivery for women with gestational diabetes (GDM) remain controversial [1, 2], with guidelines frequently suggesting elective cesarean delivery (CD) in cases of suspected macrosomia or other significant risk factors [3]. Intrapartum CD carries a higher complication rate compared to planned CD [4], regardless of the a priori increased rates of obstetric complications among women with GDM [3, 5–8]. Furthermore, women with GDM exhibit a higher incidence of non-elective CD as compared to low risk pregnancies [9–11], and increased risk for post-operative complications [12]. Therefore, it is essential to develop an efficient and reliable tool to aid physicians and patients make informed decisions and reduce maternal and neonatal morbidity.

Several studies have attempted to develop prediction models for CD among women with GDM [13, 14]. However, these studies were limited by relatively small cohort size and insufficient information on labor characteristics. Therefore, we aimed to establish an efficient prediction score model to aid physicians and patients in the decision-making process.

## Methods

We conducted a retrospective cohort study of women with GDM and a singleton pregnancy who underwent a trial of labor at a university-affiliated tertiary medical center from 2011 to 2023. Exclusion criteria included elective CD, pre-gestational diabetes, estimated fetal weight > 4000 g, previous CD, and non-viable fetuses. Our institutional protocol recommended planned CD for women with an estimated fetal weight of ≥ 4000 g, a policy that remained unchanged throughout the study period.

Women were divided into two groups based on their mode of delivery: those who underwent intrapartum CD (study group) and those who achieved vaginal delivery (control group). Maternal and obstetrical characteristics were compared between groups to identify risk factors for CD.

### Data collection

Data were obtained from our departmental electronic health records. The following demographic and obstetrical variables were recorded: maternal age, pre-gestational body mass index (BMI), height, gestational weight gain (GWG), parity, mode of conception (spontaneous versus in vitro fertilization [IVF]), glucose challenge test (GCT) levels, oral glucose tolerance test (OGTT) levels, pharmacologically treated GDM, preeclampsia, gestational age at delivery, onset of labor (spontaneous versus medical induction), oxytocin use during labor, epidural anesthesia, premature rupture of membranes (PROM), intrapartum fever ≥ 38^o^ Celsius, antibiotic treatment during labor, meconium-stained amniotic fluid (MSAF), and newborn birthweight.

### Definitions

GDM was diagnosed using Carpenter and Coustan criteria [15]. Women with a GCT value of 200 mg/dL (11.1 mmol/L) were diagnosed with GDM without the necessity of an OGTT [16]. In our institution, a diagnosis of GDM is made if at least one abnormal value is present in the oral glucose tolerance test (OGTT).

At our institution, women diagnosed with GDM are initially managed with dietary counseling and lifestyle modifications. If glycemic targets are not achieved, pharmacologic treatment with insulin or oral hypoglycemic agents is initiated, with the treatment plan tailored to the individual patient’s clinical profile, in accordance with standardized institutional protocols.

GDM was stratified as either diet treated or GDM requiring pharmacological treatment beyond dietary management, necessitating either insulin or oral agents [17].

### Statistical analysis

Univariate analyses were performed to identify differences between the groups. For continuous variables, a two-tailed unpaired Student’s *t *test or Mann–Whitney test (when variables were not normally distributed) was used to assess significance. For categorical variables, the Chi-square test or Fisher’s exact test (when appropriate) was employed.

A multivariable logistic regression model, controlling for variables that were found to be statistically different between the groups, was utilized to evaluate the impact of independent variables on the outcome. Stepwise backward elimination was used to create a multivariable model with an initial discriminatory value of *p* < 0.05 to assess for model differences that might result.

Subsequently, receiver operating characteristic (ROC) curve analysis was conducted to determine cut-off values, sensitivity, and specificity for predicting the risk of intrapartum CD. Model discrimination, risk factors, and scores were assessed using the area under the ROC curve (AUC). Each predictor in the final model was assigned a point value based on its odds ratio (OR), which were then summed to create an overall risk score. Points were calculated as the nearest rounded whole integer of the selected predictors’ OR. Finally, model performance was evaluated using ROC curves, with the AUC and 95% confidence interval calculated, and prognostic accuracy assessed against a null hypothesis (area = 0.5) using a *p *value. To assess the predictive performance of the model, we performed internal validation using a hold-out method. The dataset was randomly divided into a training set (70%) and a validation set (30%). A logistic regression model was constructed using the training data, and predicted probabilities were generated for all participants. Model performance was evaluated on the validation subset using receiver operating characteristic (ROC) analysis.

The data were analyzed using Statistical Package for the Social Sciences (SPSS) software (version 29.0; SPSS Inc., Chicago, IL, USA). A *p *value < 0.05 was considered significant. The study was approved by the local Institutional Review Board (Tel-Aviv Sourasky Medical Center IRB, protocol number 0284–0-TLV, date: June 2023).

Data analysis was performed with an unidentified database. Hence, informed consent was not required.

## Results

Overall, 146,194 women delivered in our center during the study period; of them, 17,455 (11.9%) were diagnosed with GDM. Of these, 11,305 (64.8%) met the inclusion criteria.

The intrapartum CD (study group) consisted of 676 women (6.0%), while 10,629 women (94.0%) achieved vaginal deliveries (control group).

Maternal and obstetrical characteristics are detailed in Table 1. Women in the intrapartum CD group were older and had higher rates of nulliparity, body mass index (BMI), height < 160 cm, IVF mode of conception, and greater weight gain during pregnancy. In addition, women in the study group exhibited increased rates of pathological fasting glucose, GCT > 200 mg/dl, and 3 pathological results in OGTT.

**Table 1:** Maternal characteristics for the study and control groups

Characteristic	Study group (n = 676)	Control group (n = 10,629)	*p *value
Maternal age at delivery (y), mean (SD)	34.5 (± 4.9)	33.8 (± 4.7)	** < 0.001**
Gestational age at delivery (wk), IQR (25%-75%)	39.3 (38.4–40.2)	39.4 (38.6–40.2)	0.453
Maternal age > 35 years, n (%)	293 (43.3%)	4181 (39.3%)	**0.039**
Maternal age > 40 years, n (%)	94 (13.9%)	964 (9.1%)	** < 0.001**
Nulliparity, n (%)	540 (79.9%)	4754 (44.7%)	** < 0.001**
Parity ≥ 4, n (%)	13 (1.9%)	419 (3.9%)	**0.008**
IVF, n (%)	116 (17.2%)	893 (8.4%)	** < 0.001**
Pre-gestational BMI kg/m^2^, IQR (25%-75%)	24.2 (21.5–28.0)	23.3 (10.8–26.4)	** < 0.001**
Pre-gestational BMI ≥ 30 kg/m^2^, n (%)	135 (22.6%)	1436 (15.3%)	** < 0.001**
GWG (kg), IQR (25%-75%)	12.0 (8.0–16.0)	11.0 (8.0–15.0)	** < 0.001**
GWG ≥ 15 kg, n (%)	162 (27.1%)	1684 (17.9%)	** < 0.001**
Maternal height (m), IQR (25%-75%)	1.61 (1.57–1.66)	1.63 (1.6–1.68)	** < 0.001**
Maternal height < 160 cm, n (%)	229 (36.2%)	2253 (22.8%)	** < 0.001**
Preeclampsia, n (%)	55 (8.1%)	214 (2.0%)	** < 0.001**
GDM pharmacologically treated, n (%)	55 (8.1%)	902 (8.5%)	0.751
GCT, IQR (25%-75%)	153 (141–170)	152 (141–167)	**0.004**
GCT > 200 mg/dl, n (%)	39 (5.8%)	346 (3.3%)	** < 0.001**
Fasting—OGTT, IQR (25%-75%)	87 ( 80–95)	85 (79–93)	**0.023**
Fasting—pathological OGTT, n (%)	149 (22.0%)	1968 (18.5%)	**0.007**
1-h OGTT mg/dl, IQR (25%-75%)	185 (164–202)	185 (169–199)	0.385
1-h pathological OGTT, n (%)	299 (44.2%)	4865 (45.8%)	0.436
2-h OGTT mg/dl, IQR (25%-75%)	155 (135–174)	155 (133–170)	0.113
2-h pathological OGTT, n (%)	248 (36.7%)	3794 (35.7%)	0.602
3-h OGTT mg/dl, IQR (25%-75%)	106 (80–130)	102 (77–127)	0.200
3-h pathological OGTT, n (%)	83 (12.3%)	1094 (10.3%)	0.101
2 pathological OGTT, n (%)	155 (22.9%)	2433 (22.9%)	0.981
3 pathological OGTT, n (%)	63 (8.4%)	717 (6.5%)	**0.016**
4 pathological OGTT, n (%)	10 (1.5%)	116 (1.1%)	0.352

*SD* = standard deviation*; IQR* = interquartile range; *IVF* = in vitro fertilization;* BMI* = body mass index; *GWG* = gestational weight gain; GDM = gestational diabetes; GCT = glucose challenge test; OGTT = oral glucose tolerance test

Significant differences (*p* < .05) are presented in BOLD

**Fig. 1:**
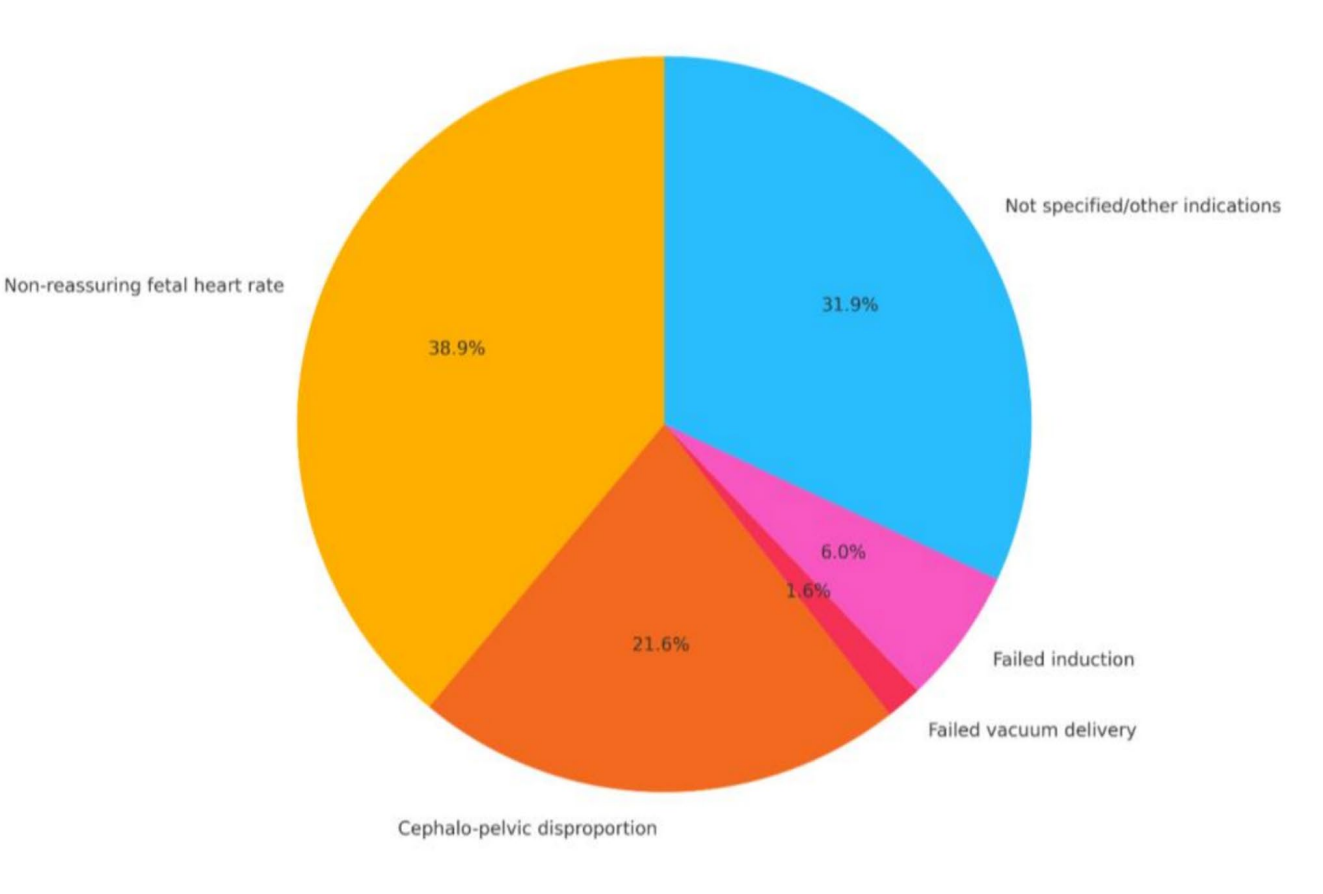
Cesarean delivery indications

Labor characteristics for the study and control groups are presented in Table 2, and the prevalence of indications for cesarean delivery is depicted in Fig. 1. Women in the intrapartum CD group had higher rates of labor induction, regional epidural anesthesia, augmentation of labor, MSAF, preeclampsia, and newborns birthweight > 3,500 g.

**Table 2:** Obstetrical characteristics of the study population during labor

Characteristic	Study group (n = 676)	Control group (n = 10,629)	*p *value
Intrapartum fever ≥ 38° (Celsius), n (%)	18 (2.7%)	240 (2.3%)	0.494
Epidural anesthesia, n (%)	591 (87.4%)	7807 (73.4%)	** < 0.001**
Induction of labor, n (%)	444 (65.7%)	3305 (31.1%)	** < 0.001**
PROM, n (%)	44 (6.5%)	631 (5.9%)	0.543
Meconium, n (%)	158 (23.4%)	1581 (14.9%)	** < 0.001**
Oxytocin, n (%)	574 (84.9%)	5550 (5.2%)	** < 0.001**
Antibiotics during delivery, n (%)	117 (17.3%)	2109 (19.8%)	0.108
Birthweight (gr), mean (± SD)	3281 (489)	3281 (431)	** < 0.001**
Birthweight > 3500 gr, n (%)	231 (34.0%)	3240 (30.5%)	**0.044**
Birthweight > 3750 gr, n (%)	100 (14.8%)	1364 (12.8%)	0.141

*SD* = standard deviation; *PROM* = premature rupture of membranes; *NA* = not applicable

Significant differences (*p* < .05) are presented in BOLD

In the multivariate analysis assessing risk factors for intrapartum CD (Table 3), several variables were found to be independently associated with intrapartum CD: maternal age ≥ 40 years, nulliparity, BMI ≥ 30 kg/m^2^, gestational weight gain > 15 kg, maternal height < 160 cm, IVF, preeclampsia, induction and augmentation of labor, MSAF, and newborn’s birth weight > 3,500 g. Epidural anesthesia was associated with reduced risk for intrapartum CD.

**Table 3:** Multivariate analysis

Characteristic	OR	95% CI	*p *value
Age > 40 years	2.0	1.5–2.7	< 0.001
Nulliparity	4.8	3.7–6.1	< 0.001
Preeclampsia	2.7	2.0–4.1	< 0.001
Induction of labor	2.9	2.4–3.5	< 0.001
Oxytocin use during labor	2.4	1.8–3.3	< 0.001
Maternal height < 160 cm	2.0	1.6–2.4	< 0.001
GWG > 15 kg	1.6	1.3–1.9	< 0.001
Birthweight > 3,500 g	1.4	1.2–1.7	< 0.001
BMI > 30 kg/m^2^	1.4	1.2–1.8	< 0.001
Meconium stained amniotic fluid	1.6	1.3–1.9	< 0.001
IVF	1.4	1.1–1.8	0.014
Epidural anesthesia	0.7	0.5–0.9	0.016
Glucose challenge test > 200 mg/dl	1.1	0.8–1.7	0.515
Fasting oral glucose tolerance test	1.1	0.9–1.4	0.266
3 pathological results in glucose tolerance test	1.0	0.7–1.4	0.895
Multiparity ≥ 4	1.0	0.5–2.1	0.907

**Fig. 2:**
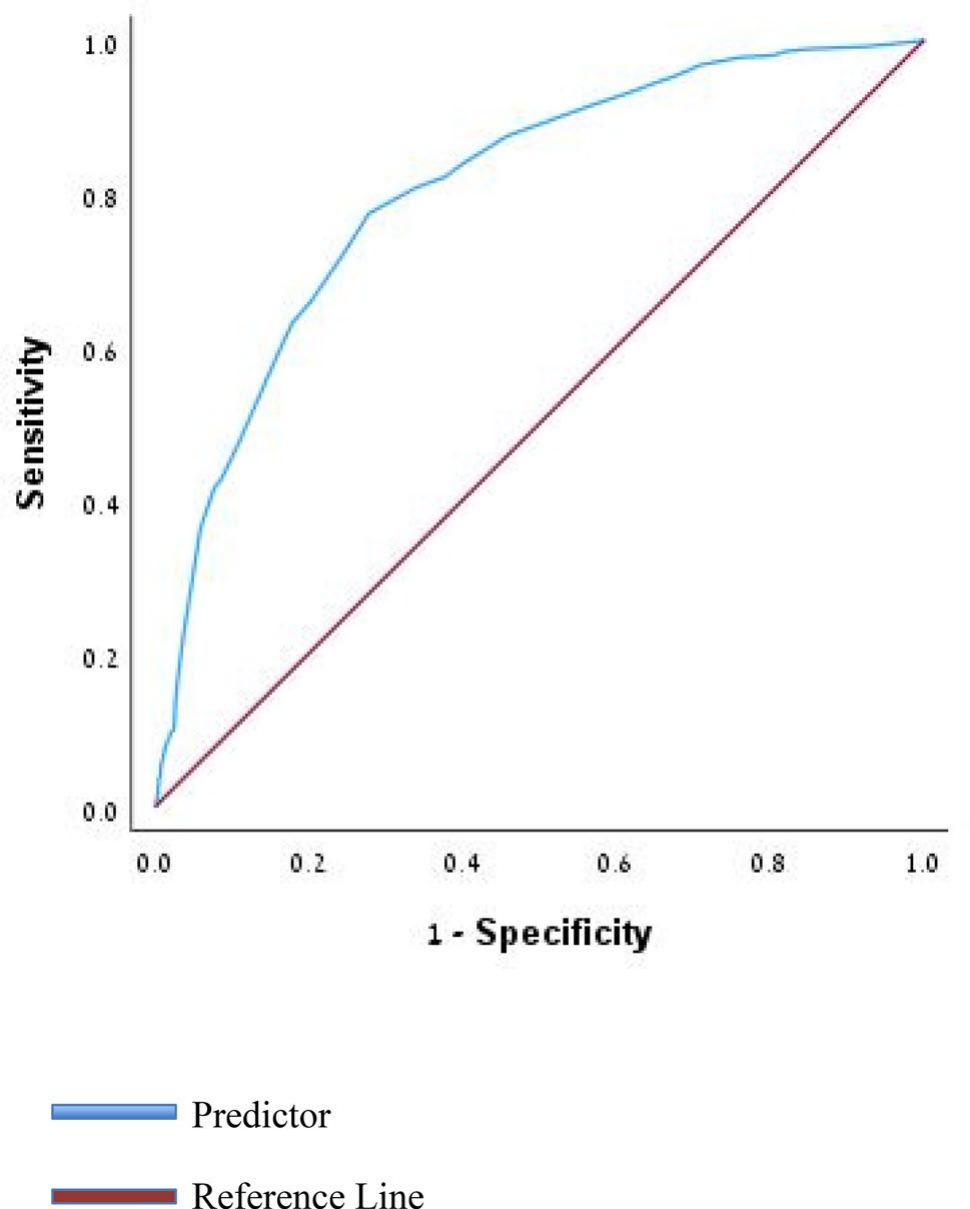
Receiver operating characteristic (ROC) curve for intrapartum cesarean delivery

*IVF* = in vitro fertilization;* BMI* = body mass index; *GWG* = gestational weight gain;

We developed a risk scoring system based on multivariable logistic regression to estimate the likelihood of intrapartum CD among women with GDM (Table 4). Patients were categorized into four risk groups, demonstrating a clear dose–response relationship: Group A (score < 4) had a 1.4% rate of intrapartum CD, Group B (score 4–8) 3.7%, Group C (score > 8 and < 14) 14.4%, and Group D (score ≥ 14) 43.1%. Model performance was evaluated using a receiver operating characteristic (ROC) curve (Fig. 2), yielding an area under the curve (AUC) of 0.807 (95% CI, 0.79–0.83).

**Table 4:** Risk of prediction score for intrapartum CD among women with GDM

Characteristic	Score
**Age > 40 years**	
Yes	2
No	0
**BMI ≥ 30**	
Yes	1.5
No	0
**Gestational weight gain ≥ 15** kg	
Yes	1.5
No	0
**Nulliparity**	
Yes	5
No	0
**Maternal height < 160 cm**	
Yes	2
No	0
**Induction of labor**	
Yes	3
No	0
**Epidural anesthesia**	
Yes	− 2
No	0
**Oxytocin during labor**	
Yes	2.5
No	0
**Meconium stained amniotic fluid**	
Yes	1.5
No	0
**Preeclampsia**	
Yes	2.5
No	0
**Birthweight ≥ 3,500 g**	
Yes	1.5
No	0
**IVF**	
Yes	1.5
No	0

The total score for each patient is calculated by summing the points assigned for each condition present. These scores correspond to defined risk groups in which patients fall into different risk categories for intrapartum CD:

Group A: < 4 points (1.4% risk)

Group B: 4—8 points (3.7% risk)

Group C: > 8 and < 14 points (14.4% risk)

Group D: ≥ 14 points (43.1% risk)

**Fig. 3:**
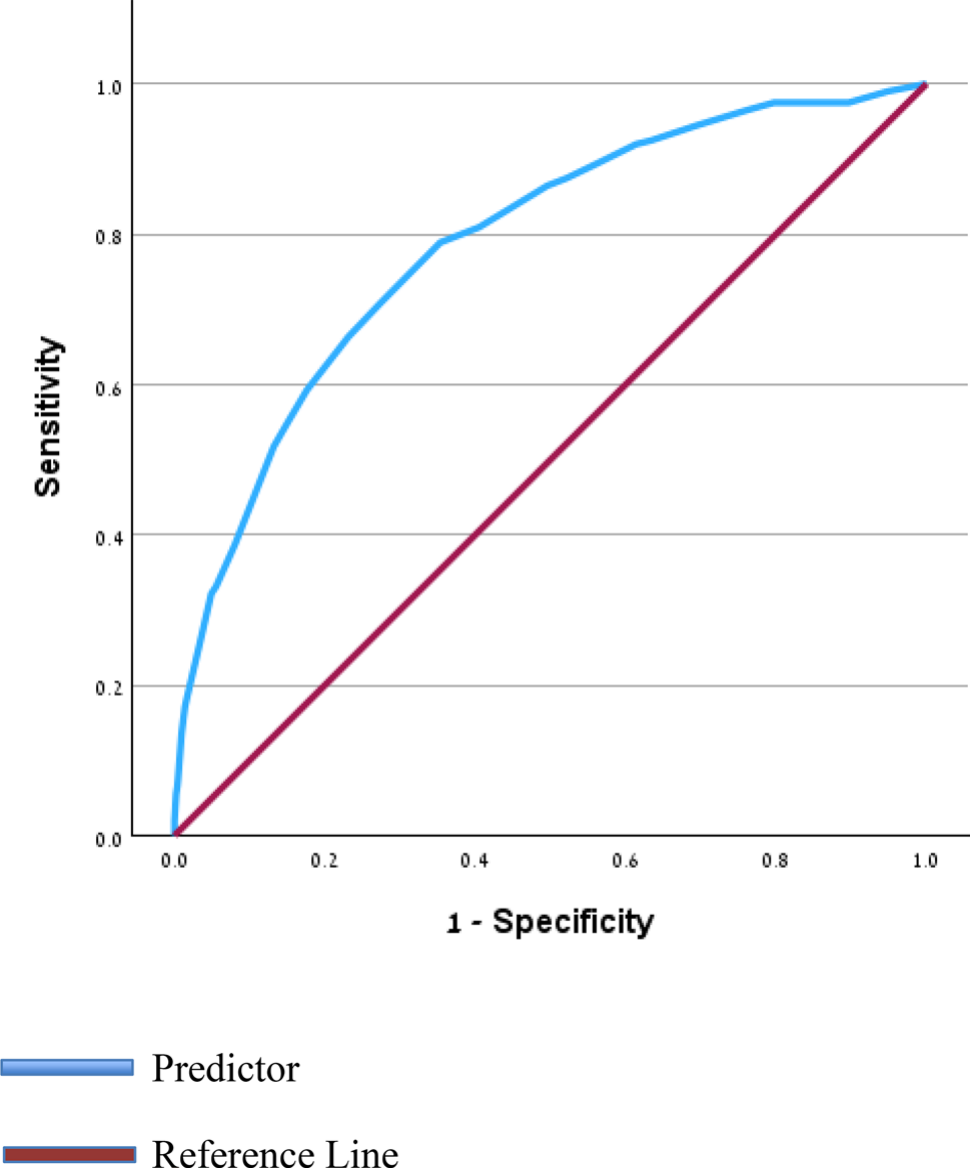
Receiver operating characteristic (ROC) curve for model performance in the validation cohort

To assess the predictive performance of the score, internal validation was conducted using a randomly selected subset comprising 30% of the cohort. On this validation set, the model demonstrated discriminative ability, with an area under the ROC curve (AUC) of 0.788 (95% CI: 0.76–0.82, *p* < 0.001) (Fig. 3).

## Comment

### Principle findings

Our study aimed to identify risk factors for intrapartum CD among women with GDM and establish a predictive model for this outcome. The main findings of our study include: [1] risk factors for intrapartum CD among women with GDM included advanced maternal age, nulliparity, increased BMI, gestational weight gain > 15 kg, maternal height < 160 cm, IVF mode of conception, induction of labor, oxytocin use during labor, preeclampsia, MSAF, and birthweight > 3,500 g; (2) epidural anesthesia was independently associated with a lower risk of intrapartum CD; (3) a prediction score model produced predictive performance with an AUC of 0.807.

### Results in the context of what is known

The global prevalence of women with GDM has been growing [18], along with their associated risk for CD [9–11, 19, 20]. Previous studies have examined the relationship between elevated BMI and gestational weight gain (GWG) in women with GDM, revealing a heightened risk for CD in those with obesity and increased GWG, consistent with our findings [20–22].

Ehrenberg et al. investigated risk factors for CD in women with GDM and corroborated our findings that labor induction, nulliparity, and BMI are significant contributors to the risk of CD. Notably, they also identified macrosomia, pharmacologically treated GDM, and being of Black ethnicity as additional risk factors [22].

Recent research has concentrated on creating predictive models for primary CD in women diagnosed with GDM. Phaloprakarn et al. [16] developed a model incorporating nulliparity, excessive GWG, and insulin treatment, while recognizing the limitations imposed by their small cohort size. Ramos et al. [13] presented a risk prediction model for intrapartum CD among women with GDM, incorporating a broader range of factors such as advanced maternal age, nulliparity, estimated fetal weight (EFW) > 90th percentile, increased BMI, preeclampsia, polyhydramnios, insulin treatment, and elevated hemoglobin A1C levels. Consistent with our findings, diagnostic levels of the OGTT did not significantly influence the risk for intrapartum CD. This study defined poor glycemic control as hospitalization for glucose stabilization among women with GDM; however, this was not identified as a significant risk factor for intrapartum CD. While their study addressed important factors, including treatment regimen and hemoglobin A1C levels, it did not incorporate labor characteristics, which may account for the enhanced predictive performance noted in our study. Both abovementioned studies identified that insulin use during pregnancy was associated with an increased risk of CD [13, 14]. However, our results did not support this finding, as the rates of pharmacologically treated GDM were comparable between the study and control groups. Further research is required to elucidate the role of pharmacological treatment on intrapartum CD among women with GDM.

While a Cochrane review found no significant impact of epidural anesthesia on CD in the general population [23], our study found that epidural anesthesia correlated with a reduced risk of intrapartum CD, notwithstanding a higher rate of epidural use in the study group. This finding aligns with a Bas-Lando’s study, which indicated that women with GDM who received epidural anesthesia experienced a reduced rate of intrapartum CD [10]. This may reflect improved maternal cooperation and labor progression under analgesia, or a selection bias in which women with early non-reassuring fetal status are triaged to cesarean delivery before labor and epidural administration, thereby excluding higher risk cases from the epidural group.

### Clinical implications

CD has become increasingly common, largely due to the rising prevalence of GDM [18]. This trend underscores the importance of selecting the appropriate delivery method for each woman to optimize clinical outcomes. Complications related to pre- and post-operative care in cases of CD are heightened by the urgency of the labor situation [4]. Therefore, conducting comprehensive pre-labor assessments and accurately predicting the risk of intrapartum CD can significantly reduce obstetric complications.

Our predictive model provides clinicians with a valuable tool for more accurately assessing the likelihood of intrapartum CD in women with GDM. The model demonstrated consistent predictive performance when applied to the independent validation cohort. These findings support the model’s reliability and its potential clinical applicability for predicting intrapartum cesarean delivery in women with GDM.

While the model incorporates key factors relevant to GDM—such as pharmacologically treated GDM and the number of pathological results from the OGTT at specific thresholds—these variables did not show a significant impact on trial of labor outcomes. This finding is noteworthy, as it suggests that these clinically relevant factors may not play a crucial role in determining the success of a trial of labor or influencing decisions regarding cesarean delivery.

Furthermore, this highlights the complex nature of labor and delivery experiences among women with GDM, indicating that factors recognized in clinical practice may not be as predictive of intrapartum outcomes as previously thought.

The implications of these considerations are both medical and economic. Careful selection of delivery methods is critical for optimizing pre-labor management and minimizing potential complications. By evaluating the predictive profile of patients and integrating both pre-labor and intra-labor characteristics, the model can greatly enrich discussions between patients and clinicians. While some variables may remain unknown to both the physician and the patient prior to a trial of labor, certain factors are particularly relevant. Ultimately, this approach aims to enhance overall patient outcomes through personalized and well-informed care strategies.

## Research implications

The predictive model for intrapartum CD among women with GDM provides a valuable tool for clinical decision-making; however, it necessitates further external validation. Future research should focus on refining this model and evaluating its effectiveness across varied patient populations to ensure its broader applicability and accuracy. By doing so, the model can be better positioned for practical use in clinical settings, ultimately enhancing patient care and outcomes.

### Strengths and limitations

The most significant limitation of this study is its retrospective design, which carries the inherent risk of selection bias, potential recording errors, and challenges in controlling for exposures and outcomes. In addition, we lacked data concerning glycemic control levels, which have been previously linked to increased risk for CD [24]. While glycemic control was not identified as a major risk factor for intrapartum CD [13], its potential role cannot be excluded.

While EFW is a known risk factor for intrapartum CD [13], it was not included due to missing data. Instead, we used neonatal birthweight as a proxy, and notably, the model still demonstrated robust performance (AUC 0.805; 95% CI 0.79–0.82), supporting the model’s resilience even in the absence of EFW data. Although the mean birthweight was identical in both groups, the standard deviation was higher in the intrapartum CD group, suggesting greater variability in fetal size among women who underwent cesarean delivery, possibly reflecting a broader range of clinical indications such as suspected macrosomia or fetal distress. In addition, the study was conducted at a single center with a relatively homogeneous population and primarily nulliparous women, potentially limiting the generalizability of the findings to other populations.

Despite its limitations, the study has several notable strengths. It includes a large cohort from a single tertiary center with uniform labor management protocols, ensuring consistency in clinical practice and reducing variability. Rigorous data collection and validation procedures enhance the study’s internal validity. The model demonstrated consistent discriminative power in internal validation (mean AUC 0.788), and it incorporates both antepartum and intrapartum variables that are routinely available in clinical settings.

Our research distinguishes itself from prior studies by incorporating both pre-labor and intra-labor characteristics, thereby improving its predictive capability and indicating a significantly higher predictive value compared to previously reported models [15, 16].

## Conclusion

We identified specific risk factors associated with intrapartum CD among women with GDM. Our analysis resulted in the development of a prediction score model exhibiting a high-performance index. This model can serve as a valuable tool for obstetricians in clinical decision-making, facilitating more informed consultations with patients regarding their delivery options. Future studies must validate these findings and refine the predictive model to improve its accuracy and applicability across various clinical settings.

## Data Availability

No datasets were generated or analyzed during the current study.
